# A family case of X-linked Alport syndrome patients with a novel variant in *COL4A5*

**DOI:** 10.1007/s13730-018-0368-4

**Published:** 2018-10-06

**Authors:** Yasuyo Kashiwagi, Shinji Suzuki, Kazushi Agata, Yasuyuki Morishima, Natsuko Inagaki, Hironao Numabe, Hisashi Kawashima

**Affiliations:** 1 Department of Pediatrics, Tokyo Medical University, 6-7-1 Nishishinjuku, Shinjuku-ku, Tokyo, Japan; 2 Clinical Genetics Center, Tokyo Medical University, 6-7-1 Nishishinjuku, Shinjuku-ku, Tokyo, Japan

**Keywords:** Alport syndrome (AS), X-linked, *COL4A5*, RAAS blockade

## Abstract

We herein report 2 Japanese patients with X-linked Alport syndrome (XLAS), with a novel variant in *COL4A5*. Patient 1 was a 16-year-old Japanese girl with a history of microscopic hematuria, without proteinuria, renal dysfunction, deafness, or ocular abnormalities. At 13 years of age, renal biopsy was performed; however, a diagnosis of AS was not considered. When her mother (patient 2) was 40 years of age (3 years after patient 1 underwent a renal biopsy), patient 2 was found to have asymptomatic hematuria, proteinuria, and an increased serum creatinine level, without deafness and ocular abnormalities. Subsequently, immunofluorescence staining for alpha 5 chains of type IV collagen was performed in patient 1. Pathological findings were consistent with AS, and genetic analysis demonstrated that both patients had a heterozygous mutation in *COL4A5* (NM_000495.4: exon41:c.C3769T: p.Q1257X). To date, more than 900 different *COL4A5* mutations have been identified; however, this variant has not been previously described. Physicians have to consider AS when they perform a renal biopsy in all patients with hematuria despite absent/present of family history, hearing loss, and ocular abnormality. Especially, when findings of light microscopy and immunofluorescence microscope are unclear, it should be considered carefully. Electron microscopy findings are very important.

## Introduction

Alport syndrome (AS) is characterized by progressive renal disease, sensorineural hearing loss, and ocular abnormalities [1]. AS is caused by the presence of abnormal alpha chains that normally form type IV collagen, which is important in basement membranes in the glomerulus, cochlea, cornea, lens, and retina [2]. The mode of inheritance of AS is considered to be X linked, autosomal recessive, and autosomal dominant, and X-linked AS (XLAS) is the most common. We herein report 2 Japanese patients with XLAS, with a novel variant in *COL4A5*.

## Case report

**Fig. 1:**
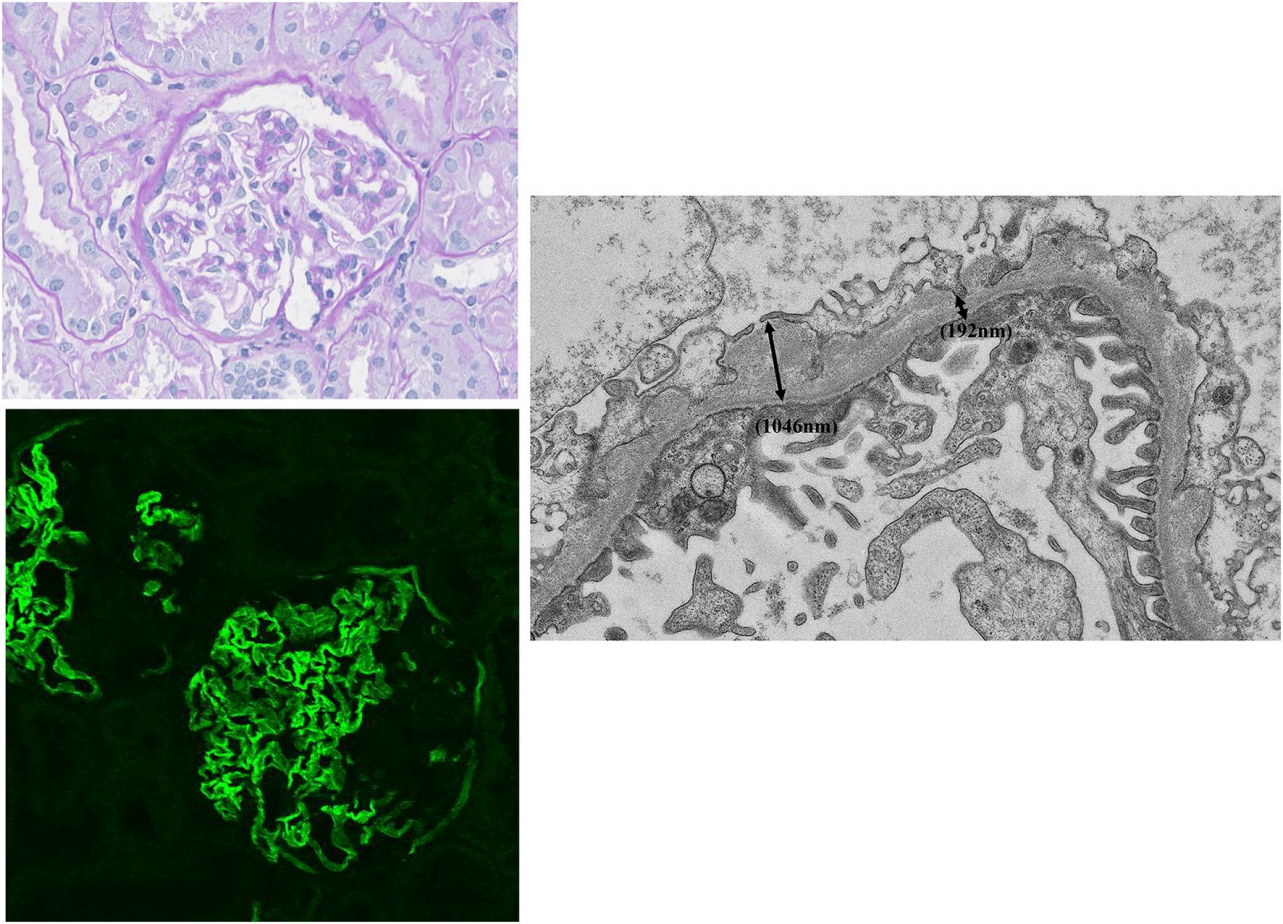
Morphological analysis of renal biopsy specimens from patient 1. PAS staining (upper left: Magnification × 400) shows mild mesangial proliferation. Immunostaining for collagen alpha 5 chains of type IV collagen (lower left) shows segmental and mosaic patterns in the GBM and Bowman’s capsule, suggesting that X-linked AS. EM (right) shows irregular thickening (maximum: 1.046 nm) and thinning (minimum: 192 nm) of the GBM (black two-way arrows) (Magnification × 4000)

The patient (patient 1) was a 16-year-old Japanese girl who was the first child of healthy nonconsanguineous parents. At 3 years of age, she was referred to our hospital because of microscopic hematuria without proteinuria. She appeared healthy with a normal weight and height for her age. She had no clinical hearing loss or ocular abnormalities. A urinalysis showed 3 + hematuria with urine sediment containing more than 100 red blood cells per high-power field. Blood laboratory results, such as blood urea nitrogen level, serum creatinine level, immunoglobulin levels, complement quantification level, and autoantibodies were normal. Renal ultrasonography was unremarkable. At 13 years of age, a renal biopsy was performed to be suspected IgA nephropathy, because, at that time, there were sometimes gross hematuria attacks when she caught cold. Periodic acid–Schiff (PAS) staining (Fig. 1, upper left) showed mild mesangial proliferation. Other findings of the tubules and interstitium showed no significant alterations. Staining of IgA in immunofluorescence (IF) was negative; histopathological and clinical diagnosis at biopsy was non IgA nephropathy. The findings of electron microscopy (EM) were not evaluated at this time.

The mother of patient 1 (patient 2) was healthy without renal dysfunction, deafness, or ocular abnormalities. However, at 40 years of age (3 years after patient 1 underwent the renal biopsy), she was referred to our hospital for chance proteinuria and chance hematuria. Her urinalysis showed 2 + proteinuria (P/Cre 2.3 g/gCr) and 1 + hematuria with urine sediment containing 5–9 red blood cells per high-power field. Her blood urea nitrogen level, serum total protein level, and complement quantification level were normal; however, she had an increased serum creatinine level (1.57 mg/dL). Patient 2 showed atrophy right kidney when she was referred to our hospital, so we could not do her renal biopsy.

Subsequently, we re-evaluated the EM and IF data for alpha 5 chains of type IV collagen in patient 1 (antibody H53; Shigei Medical Research Institute, Okayama, Japan). EM demonstrated irregular thickening (maximum: 1,046 nm) and thinning (minimum: 192 nm) of the glomerular basement membrane (GBM; Fig. 1, right). IF staining for alpha 5 chains of type IV collagen showed segmental and mosaic patterns in the GBM and Bowman’s capsule (Fig. 1: lower left).

After the whole-exome sequencing study was approved by the Ethics Committee of Tokyo Medical University, written informed consent was obtained from the patient and both of her parents.

Peripheral blood mononuclear cells were isolated from 5 mL of whole blood with Ficoll-Paque™ PLUS (GE Healthcare), and genomic DNA was extracted using QIAamp® DNA Blood Mini Kit (Qiagen). Exome enrichment and library preparation were performed with Ion Ampliseq™ Exome RDY Kit PI v3 (Thermo Fisher Scientific) and Ion Xpress™ Barcode Adapters (Thermo Fisher Scientific). Library concentration was measured with Ion Library™ TaqMan Quantitation Kit (Thermo Fisher Scientific). After template preparation using Ion PI™ Hi-Q™ Chef kit (Thermo Fisher Scientific) on the Ion Chef™ system (Thermo Fisher Scientific), sequencing was performed with the Ion Proton™ sequencer (Thermo Fisher Scientific). VCF files were generated from the sequence data using the Torrent Suite™ software on the Torrent Server. All parameters were used at the default settings. VCF files were annotated with ANNOVAR.

Deleterious variants were filtered using the following settings. Variants on exon or splice sites of Alport syndrome-causing genes (*COL4A3, COL4A4*, and *COL4A5*), an allele frequency of less than 1% in the Human Genomic Variation Database or the 1000 genome project, were not observed in in-house data. Validation of the results was performed by the standard Sanger sequencing techniques using with the ABI PRISM 310 Genetic Analyzer.

**Fig. 2:**
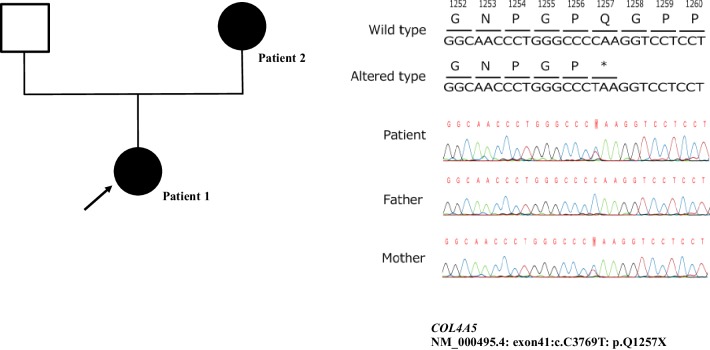
Family tree showing the distribution of genomic variants. The arrow indicates the proband. Individuals without kidney diseases are denoted by empty squares (father). Affected individuals with X-linked AS are denoted by black circles (patients 1 and 2). The proband and her mother have the same variant, c.C3769T in *COL4A5* exon 41 (NM_000495.4: exon41:c.C3769T: p.Q1257X). Her father does not have this variant

Patients 1 and 2 were found to have a heterozygous variant in *COL4A5* (NM_000495.4: exon41:c.C3769T: p.Q1257X), whereas the father of patient 1 did not have this variant (Fig. 2).

## Discussion

*COL4A5* mutations are associated with the major X-linked form of the disease, and *COL4A3* and *COL4A4* mutations are associated with autosomal recessive, dominant forms, and benign familial hematuria. The introduction of next-generation sequence allowed to improve mutation screening in familial hematuric nephropathies [3, 4]. In men with XLAS who show a typically severe phenotype, the GBM becomes fragile, leading to end-stage renal disease; however, the degree of the phenotypes in XLAS women is variable [5, 6]. Because of the presence of 2 X chromosomes, female carriers of XLAS typically have a less severe phenotype than male patients [6] and the diagnosis of XLAS is often not considered. In the present case, patients 1 and 2 had no clinically detectable hearing loss or ocular abnormalities. We suspected XLAS in patient 1 after her mother (patient 2) demonstrated asymptomatic hematuria, proteinuria, and an increased serum creatinine level. If at biopsy the findings of EM in patient 1 were evaluated adequately, a diagnosis of AS might be suggested earlier. Although microscopic hematuria was noted earlier in patient 1, it took a long time to diagnose her as having XLAS.

Cases of XLAS are caused by variants in the *COL4A5* gene encoding the type IV collagen alpha 5 chain. Collagen type IV alpha 5 has a 14-amino-acid signal peptide, followed by a 1,414-amino-acid collagenous domain, and a 224-amino-acid C-terminal NC1 domain. The C-terminal NC1 domain is involved in the alignment of individual alpha chains into a triple-helical structure. The variant identified in this case was c.C3769T: p.Q1257X, resulting in a glutamine to stop codon at position 1257 in exon 41. The clinical interpretation guidelines of the American College of Medical Genetics and Genomics [7] have classified this variant as pathogenic. This variant is expected to result in truncated collagen monomers without the NC1 domain, leading to malformation of the triple-helix structure of collagen IV. More than 900 COL4A5 mutations have been reported to date in HGMD database; however, the variant identified in this study has not been previously described.

It has been suggested that, for the treatment of AS, renin–angiotensin–aldosterone system (RAAS) blockade should be initiated before renal function is impaired [8]. Gross et al. demonstrated that the best therapeutic effect was obtained in AS patients when the treatment was initiated before the development of proteinuria [9]. An excessive amount of proteins in the glomerular filtrate activate proinflammatory and profibrotic signaling pathways in proximal tubular epithelial cells, leading to deterioration of renal function. In this case, recently, patient 1 show mild proteinuria, so we treat both patients with RAAS blockade (ACE I) and pay close attention to their clinical course.

Differences in X-chromosome inactivation may affect disease severity. Abe et al. identified a novel variation in a Japanese family using X-chromosome inactivation assays [10]. Yamamura et al. demonstrated that the phenotype of female XLAS patients is not always mild, and the mechanisms determining the severity of female XLAS are multifactorial [11].

Physicians have to consider AS when they perform a renal biopsy in all patients with hematuria despite absent/present of family history, hearing loss, and ocular abnormality. Especially, when findings of light microscopy and IF microscope are unclear, it should be considered carefully. EM findings are very important.
